# Diagnosis and ECMO treatment of a critically ill patient with heart attack: a case report

**DOI:** 10.1093/omcr/omae205

**Published:** 2025-03-20

**Authors:** Xuan Zhang, Yiping Deng, Xinlong Liu

**Affiliations:** Department of Emergency, Shenzhen Guangming District People's Hospital, Shenzhen, China; Department of Emergency, Shenzhen Guangming District People's Hospital, Shenzhen, China; Department of Emergency, Shenzhen Guangming District People's Hospital, Shenzhen, China

**Keywords:** extracorporeal membrane oxygenation, acute myocardial infarction, cardiogenic shock, percutaneous coronary intervention, sudden cardiac death

## Abstract

We report a case of a 49-year-old male diagnosed with acute myocardial infarction (AMI). After continuous cardiopulmonary resuscitation (CPR), percutaneous coronary intervention (PCI), and treatment with continuous renal replacement therapy (CRRT), extracorporeal membrane oxygenation (ECMO) and mechanical ventilation, the patient's condition improved well without sequela. This case report will demonstrate how ECMO and intensive care was used to acute myocardial infarction and cardiogenic shock.

## Introduction

AMI remains a leading cause of morbidity and mortality worldwide, necessitating prompt recognition and effective treatment strategies. Early interventions, as PCI and thrombolytic therapy, are crucial in restoring coronary blood flow and preventing irreversible myocardial damage [1].

In China, cardiovascular diseases is a leading cause of mortality. In 2020, 24.28 million Chinese people was in hospital due to cardiovascular diseases, and up to 32.90% diagnosed with ischemic heart disease [2]. Ischemic heart disease always lead to cardiac arrest (CA). Venoarterial ECMO (VA-ECMO) is employed in cases of cardiogenic shock, providing both oxygenation and circulatory support [3].

In this case report, we report a case of CA not responding well to normal resuscitation protocol. The patient was successfully resuscitated after a period of support with VA-ECMO. We aim to demonstrate the effectiveness of ECMO as a bridge to recovery and emphasize the importance of timely intervention in optimizing patient outcomes.

## Case report

**Figure 1:**
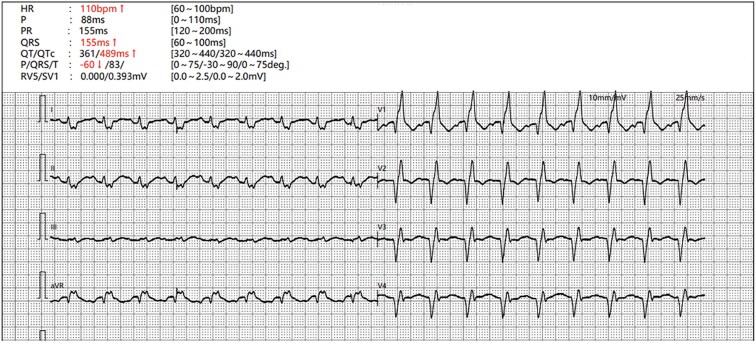
ECG on patient's arrival.

A 49-year-old long-term-smoke male was transferred to the emergency department of our hospital unconsciously, who got rounds of CPR and electric defibrillation in ambulance. At the time arriving in our hospital at 15:24 9^th^ May, the patients’ heart rate was 104 bpm, systolic blood pressure was 130 mmHg, diastolic blood pressure was 99 mmHg with noradrenaline (2.0 μg/kg/min) and dobutamine (15.0 μg/kg/min) support, and oxygen saturation was 85% with 6 l/min oxygen supply. Laboratory data demonstrated serum aspartate aminotransferase (995 IU/l), alanine aminotransferase (315 IU/L), creatinine (171.7 μmol/l), lactate (19.0 mmol/l), NT-pro brain natriuretic peptide (BNP) (406 pg/ml), and troponin T (59.7 ng/ml). The blood gas analysis revealed pH 7.008, pCO2 41.7 mmHg, pO2 69.3 mmHg, AB 10.5 mmol/l, and BE −20.2 mmol/l. And the electrocardiogram (ECG) shows as follows (Fig. 1). After intravenously injecting 1 mg adrenalin every 3 min, which sum to 34 times, along with CPR, mechanical ventilation, cardiotonic, and diuretic treatment, the patient recovered to spontaneous heartbeat at 15:34 and transferred to intensive care of unit (ICU) at 19:40.

With the treatment of respiratory support, vasopressor drugs, anti-infection rescue, CRRT and so on, his vitals gradually stabilized. In the 02:10 10^th^ May, VA-ECMO was introduced, A venous drainage cannula (21 Fr) was inserted into the left femoral vein and an arterial return cannula (15 Fr) into the right femoral artery. The blood flow rate of ECMO was maintained 3.0 l/min and 100% oxygen was given at 3.0 l/min.

**Figure 2:**
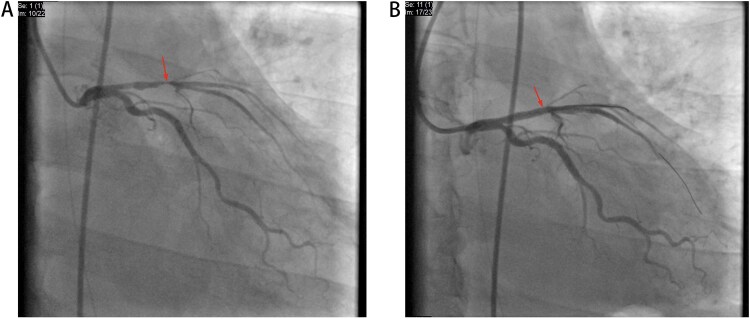
(A) At the start of CAG. (B) After PCI.

**Figure 3:**
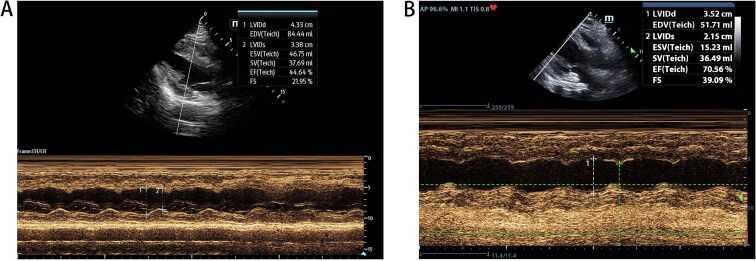
(A) Echocardiography of 10th May, EF = 44.64%. (B) Echocardiography of 18th May, EF = 70.56%.

**Figure 4:**
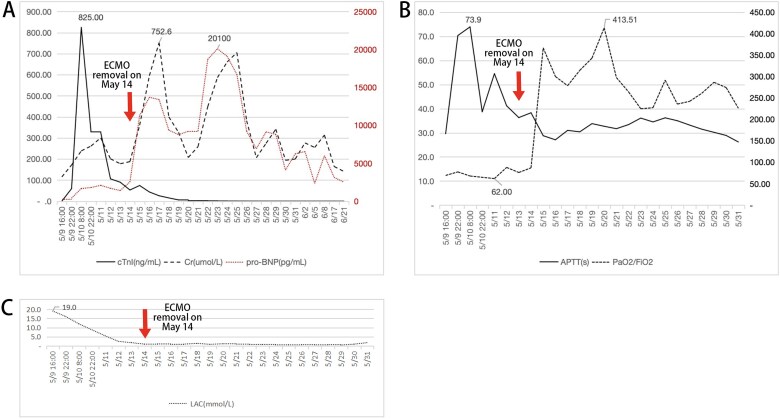
Continuous test recorded. About cTnl, Cr and pro-BNP (A), APTT and ratio P/F (B) and LAC (C). These charts showed a serious multi-organ dysfunction occured in the patient and he recovered within a short time.

Then at 08:50, coronary angiography (CAG) and PCI was operated (Fig. 2). With time went on, his status improved well and in the 19:00 14^th^ May, VA-ECMO was withdrawn. Echocardiography showed the patient’s heart function improved (Fig. 3) and laboratory data indicted that he had a good recovery (Fig. 4). In the 21^st^ May, ventilator was withdrawn, and in the 31^st^ May, he was transferred from ICU to the Department of Cardiology.

With treatment of 3 more weeks, the patient left hospital without chest pain, chest distress or any other discomforts. He came to the clinic in late June that showed a stable condition. Now he could care for himself without sequela.

## Discussion

In this case report, we presented a critically ill patient with AMI who was successfully treated with VA-ECMO. The major findings demonstrated that timely initiation of ECMO effectively stabilized the patient, improved cardiac function, and allowed for subsequent intervention through PCI. The patient experienced a significant reduction in vasoactive medication requirements, reflecting enhanced hemodynamic stability during ECMO support. This aligns with existing literature that underscores the critical role of ECMO in managing severe cardiac dysfunction, particularly in cases where conventional therapies fail.

The literature supports the assertion that early initiation of ECMO in patients with cardiogenic shock can lead to better outcomes [4]. Studies have shown that patients receiving ECMO within the first few hours of cardiogenic shock onset demonstrate improved survival rates compared to those who are delayed in ECMO initiation [5].

However, there are limitations to this case report that should be acknowledged. First, this is a single-case analysis, which may limit the generalizability of the findings. Additionally, the complexities of ECMO management, including the risk of complications such as bleeding and infection, were not fully explored in this case. Future studies involving larger cohorts and randomized controlled trials are necessary to better understand the long-term outcomes and potential complications associated with ECMO in patients with acute myocardial infarction and cardiogenic shock.
